# Influence of Various Strontium Formulations (Ranelate, Citrate, and Chloride) on Bone Mineral Density, Morphology, and Microarchitecture: A Comparative Study in an Ovariectomized Female Mouse Model of Osteoporosis

**DOI:** 10.3390/ijms25074075

**Published:** 2024-04-06

**Authors:** Agnieszka Tomczyk-Warunek, Karolina Turżańska, Agnieszka Posturzyńska, Filip Kowal, Tomasz Blicharski, Inés Torné Pano, Anna Winiarska-Mieczan, Anna Nikodem, Sławomir Dresler, Ireneusz Sowa, Magdalena Wójciak, Piotr Dobrowolski

**Affiliations:** 1 Laboratory of Locomotor Systems Research, Department of Rehabilitation and Physiotherapy, Medical University of Lublin, 20-954 Lublin, Poland; agnieszka.tomczyk-warunek@umlub.pl; 2 Department of Orthopaedics and Rehabilitation, Medical University of Lublin, 20-954 Lublin, Poland; agnieszka.posturzynska@umlub.pl (A.P.); reh-ortop@umlub.pl (F.K.); tomasz.blicharski@umlub.pl (T.B.); itornepano@gmail.com (I.T.P.); 3 Department of Bromatology and Nutrition Physiology, Institute of Animal Nutrition and Bromatology, University of Life Sciences in Lublin, Akademicka St. 13, 20-950 Lublin, Poland; anna.mieczan@up.lublin.pl; 4 Department of Mechanics, Materials and Biomedical Engineering, Faculty of Mechanical Engineering, Wrocław University of Science and Technology, Wybrzeże Wyspiańskiego, 50-370 Wrocław, Poland; anna.nikodem@pwr.edu.pl; 5 Department of Analytical Chemistry, Medical University of Lublin, 20-093 Lublin, Poland; slawomir.dresler@umlub.pl (S.D.); ireneusz.sowa@umlub.pl (I.S.); magdalena.wojciak@umlub.pl (M.W.); 6 Department of Plant Physiology and Biophysics, Institute of Biological Science, Maria Curie-Skłodowska University, 20-033 Lublin, Poland; 7 Department of Functional Anatomy and Cytobiology, Maria Curie-Skłodowska University, 20-033 Lublin, Poland; piotr.dobrowolski@umcs.lublin.pl

**Keywords:** osteoporosis, strontium ranelate, strontium chloride, strontium citrate, bone, strontium supplementation

## Abstract

Osteoporosis stands out as a prevalent skeletal ailment, prompting exploration into potential treatments, including dietary strontium ion supplements. This study assessed the efficacy of supplementation of three strontium forms—strontium citrate (SrC), strontium ranelate (SrR), and strontium chloride (SrCl)—for enhancing bone structure in 50 female SWISS mice, aged seven weeks. In total, 40 mice underwent ovariectomy, while 10 underwent sham ovariectomy. Ovariectomized (OVX) mice were randomly assigned to the following groups: OVX (no supplementation), OVX + SrR, OVX + SrC, and OVX + SrCl, at concentrations equivalent to the molar amount of strontium. After 16 weeks, micro-CT examined trabeculae and cortical bones, and whole-bone strontium content was determined. Results confirm strontium administration increased bone tissue mineral density (TMD) and Sr content, with SrC exhibiting the weakest effect. Femur morphometry showed limited Sr impact, especially in the OVX + SrC group. This research highlights strontium’s potential in bone health, emphasizing variations in efficacy among its forms.

## 1. Introduction

Osteoporosis (OP) is currently the most common skeletal system disease globally [1]. This condition causes disturbances in the metabolic processes of the bone tissue, therefore contributing to the increase in osteolytic over osteogenic processes, effectively impairing the microstructure and decreasing bone mineral density (BMD) [1,2]. Osteoporosis develops asymptomatically for a long time. The first symptom is usually a bone fracture [1,2,3]. Osteoporosis can develop in individuals of any age, irrespective of gender and ethnicity. However, the predominant forms are postmenopausal and senile osteoporosis. With the aging of the population and the rise in average age, osteoporosis is emerging as a worldwide epidemic [2]. Globally, 22.1% of women and 6.6% of men over the age of 50 suffer from osteoporosis [3]. As a result of OP, almost 9 million fractures occur worldwide. These fractures put a significant strain on the healthcare system. In the EU (*European Union*) alone, it is estimated that in the population aged 50–84, approximately 22 million women and 5.5 million men suffer from osteoporosis [1,4].

According to the data of the National Health Fund in Poland, in 2018, 1.7 million women and 0.4 million men suffered from osteoporosis [5]. In 2018, the reimbursement of benefits provided due to osteoporosis was PLN 42 million and the reimbursement of drugs was PLN 47.6 million (National Health Fund Osteoporosis Report 2019). The cost of treating OP fractures is estimated at EUR 37 billion. It is forecasted that in 2025, the cost of health care for patients with OP will increase by as much as 25% [1].

Despite the ever-expanding understanding of the disease’s nature, osteoporosis treatment is a constant challenge. One of the currently proposed solutions is dietary supplements containing strontium ions, which significantly affect the process of bone tissue remodeling. Strontium ions are incorporated in the bone in a similar manner as calcium, improving bone structural parameters [6]. However, the availability of this element varies depending on the salt concentration [7]. Currently, the pharmaceutical market offers many such preparations. This group of pharmaceuticals includes strontium citrate (SrC) and strontium chloride (SrCl). The action of these compounds is compared to the effect of strontium ranelate (SrR) on bone tissue, a drug that simultaneously stimulates osteogenesis processes and inhibits osteolysis processes [8,9,10]. Despite the effectiveness of SrR in OP treatment, its use has currently been limited or withdrawn in numerous countries, due to the occurrence of numerous complications, including an increased risk of deep vein thrombosis, especially in patients with a high risk of stroke and ischemic heart disease [11]. However, due to its action, this drug, in its generic form, has been used in the UK again since 2019 [12].

In this study, the efficacy of supplementation with strontium citrate and strontium chloride, in comparison with strontium ranelate, in enhancing bone structural parameters was evaluated. A mouse model with induced osteoporosis by ovariectomy was used as a model in the experiment. Animals received equivalent molar doses of Sr in the form of different salts. The total content of elements essential for bone formation (strontium, calcium, and phosphorus) was assessed using inductively coupled plasma—optical emission spectroscopy (ICP—OES). A computerized microtomography (micro—CT) was applied to investigate the effects of supplementation on bone tissue mineral density (TMD), as well as the morphology and morphometry of the trabecular and cortical bones [13,14]. Micro-CT provides more reliable results than densitometry (DXA), which is regarded as the “gold standard”. In studies evaluating the effect of strontium administration on BMD value, which encompasses both bone and bone marrow mineral density, the BMD value may be distorted when using DXA. Sr is characterized by a higher atomic mass than calcium, which contributes to the overestimation of the BMD value. Various studies have shown that incorporating 1% of strontium into bone increases BMD by 10% when DXA was used [7]. Also, the DXA test only gives a preview of the BMD of the whole bone, without the division into cortical and cancellous bone tissue. On the other hand, the micro-CT examination is more accurate because it is based on a 3D reconstruction consisting of several hundred 2D images. Also, this technique enables the determination of TMD separately for trabeculae and cortical bones.

## 2. Results

### 2.1. The Body Weight of the Animals

**Figure 1:**
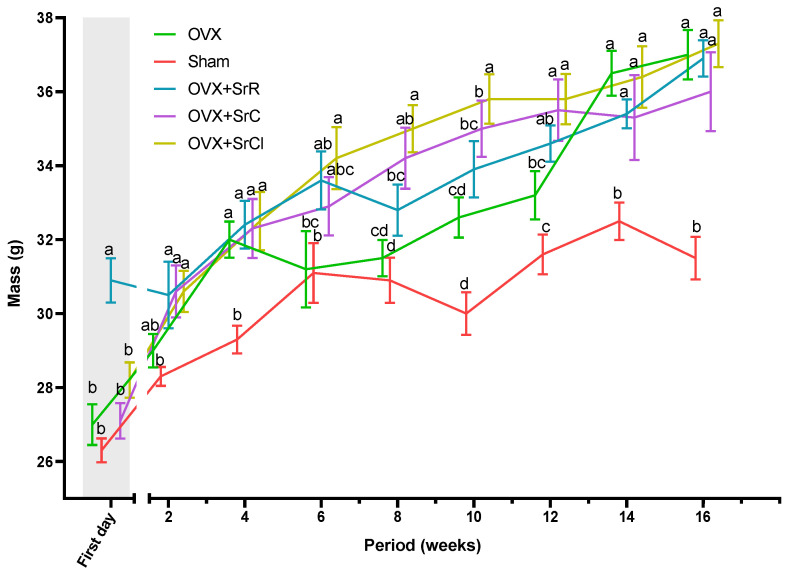
Effect of SrR, SrC, and SrCl administration on body weight in mice with induced osteoporosis on subsequent days of the study after OVX or sham. The data given are the mean (n = 10) with the standard error. The values with different letters differ significantly at *p* < 0.05. OVX—ovariectomy group without supplementation, sham—sham ovariectomy group without supplementation, OVX + SrR—ovariectomy + strontium ranelate administration; OVX + SrC—ovariectomy + strontium citrate group; OVX + SrCl—ovariectomy + strontium chloride group.

After 16 weeks of the experiment, a statistical examination revealed a significant difference in body weight between the OVX group and the sham group. Body weight gain in the OVX group was approximately 37%, while in the sham group, it was approximately 19.8%. After the experiment, the body weight of animals in all three experimental groups receiving strontium salts did not significantly differ from that of animals in the OVX group. The body weight gain in the OVX + SrR group was lower than in the OVX and the OVX + SrC and OVX + SrCl groups (Figure 1).

### 2.2. Osteometric Properties of the Femur

**Figure 2:**
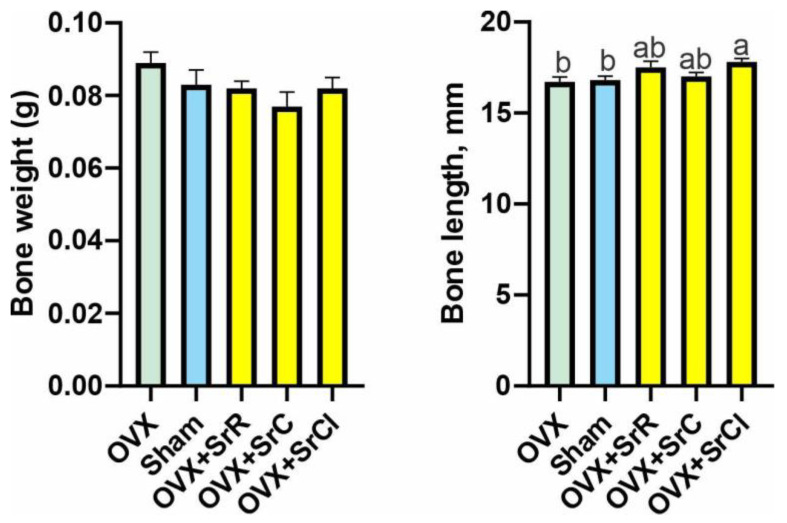
Effect of SrR, SrC, and SrCl administration on the osteometric properties of the femur in mice with induced osteoporosis. The data given are the mean (n = 10) with the standard error. The values with different letters differ significantly at *p* < 0.05. OVX—ovariectomy group without supplementation; sham—sham ovariectomy group without supplementation; OVX + SrR—ovariectomy + strontium ranelate; OVX + SrC—ovariectomy + strontium citrate; OVX + SrCl—ovariectomy + strontium chloride.

There was no effect of SrR, SrC, or SrCl administration on the femur weight of the tested animals in comparison to the OVX—ovariectomy group without supplementation—and sham—sham ovariectomy group without supplementation—groups. There were also no differences in bone mass between the study groups and both controls (Figure 2). The femur length was significantly longer in the OVX + SrCl group compared to the OVX and sham groups. Differences in bone length were not observed when comparing all three strontium supplemented groups (groups OVX + SrR, OVX + SrC, and OVX + SrCl). Statistical analysis showed no differences in femur length between the OVX and sham groups (Figure 2).

### 2.3. Bone Mineral Density of the Trabecular and Cortical Bones

**Figure 3:**
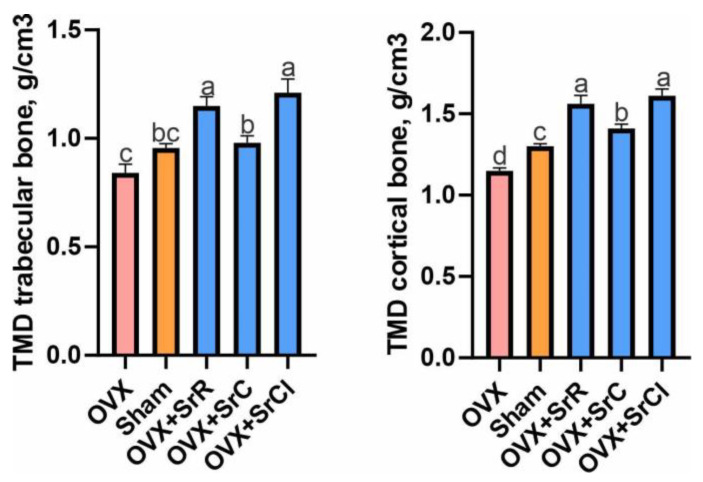
Effect of SrR, SrC, and SrCl administration on the tissue mineral density (TMD) of the trabecular and cortical bone of the femur in mice with induced osteoporosis. The data given are the mean (n = 10) with the standard error. The values with different letters differ significantly at *p* < 0.05. OVX—ovariectomy group without supplementation; sham—sham ovariectomy group without supplementation; OVX + SrR—ovariectomy + strontium ranelate; OVX + SrC—ovariectomy + strontium citrate; OVX + SrCl—ovariectomy + strontium chloride.

Statistical analysis showed that the administration of SrR and SrCl in animals contributed to a significant increase in the mineral density of the trabecular bone compared to the sham group. However, compared to the OVX group, all groups receiving strontium salts had a significantly higher TMD. In the groups receiving SrR, SrC, and SrCl, the group receiving SrC had a significantly lower cancellous bone tissue mineral density compared to the other experimental groups (Figure 3).

Animals from the sham group had a significantly higher TMD of the cortical bone compared to animals from the OVX group. Also, the statistical study showed that animals receiving strontium salts had a significantly higher cortical bone mineral density compared to both the OVX and sham groups. However, SrC administration resulted in the lowest increase in cortical bone mineral density of all the strontium salts tested (Figure 3).

### 2.4. Cancellous Bone Microarchitecture

**Figure 4:**
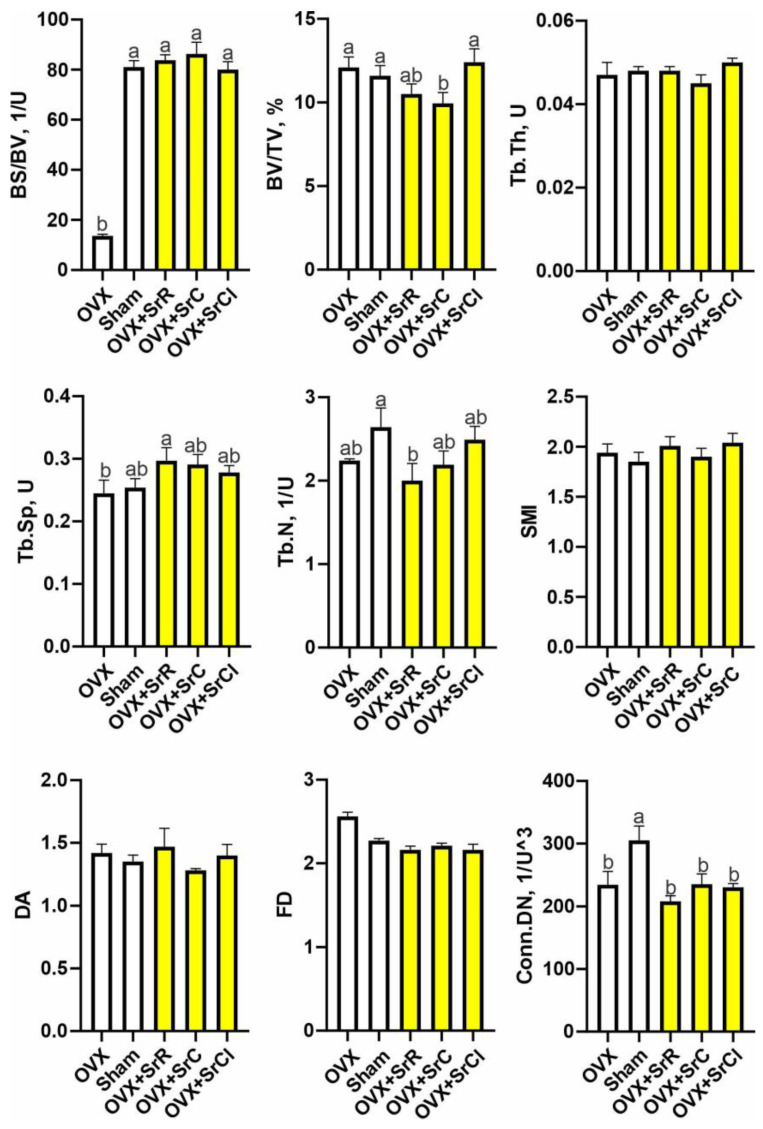
Effect of SrR, SrC, and SrCl administration on the trabecular bone microarchitecture of the femur in mice with induced osteoporosis, calculated based on micro-CT images. The data given are the mean (n = 10) with the standard error. The values with different letters differ significantly at *p* < 0.05. OVX—ovariectomy group without supplementation; sham—sham ovariectomy group without supplementation; OVX + SrR—ovariectomy + strontium ranelate; OVX + SrC—ovariectomy + strontium citrate; OVX + SrCl—ovariectomy + strontium chloride. BS/BV—specific bone surface; BV/TV—percent bone volume; Tb.Th—trabecular thickness; Tb.sp—trabecular separation; Tb.N—trabecular number; SMI—structure model index; DA—degree of anisotropy; FD—fractal dimension; Conn.Dn—connectivity density.

**Figure 5:**
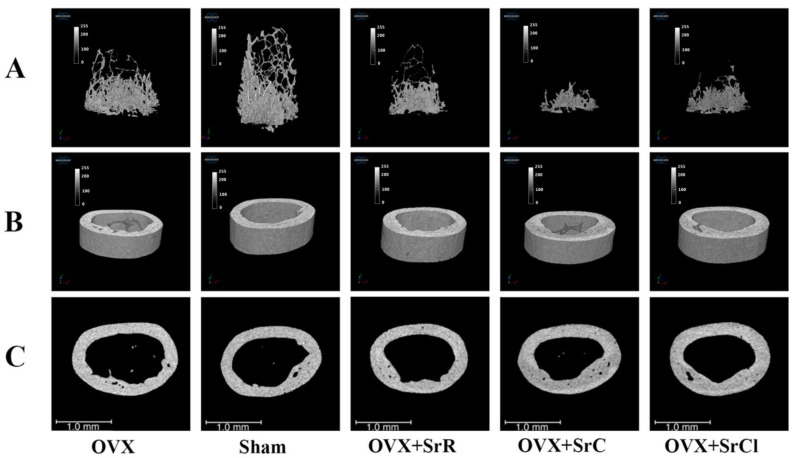
Representative micro-CT images of the 3D reconstruction and the transverse plane of the cortical femurs of mice with induced osteoporosis after SrR, SrC, and SrCl administration. (**A**): bone trabeculae; (**B**,**C**): cortical bone. OVX—ovariectomy group without supplementation, sham—sham ovariectomy group without supplementation, OVX + SrR—ovariectomy + strontium ranelate administration; OVX + SrC—ovariectomy + strontium citrate group; OVX + SrCl—ovariectomy + strontium chloride group.

Micro-CT analysis of the femoral trabecular bone’s microarchitecture showed that the sham group had higher values of BS/BV and Conn.Dn than the OVX group (Figure 4). The administration of SrR in animals with induced osteoporosis contributed to a decrease in the values of the following parameters: BV/TV, Tb.N, and Conn.Dn, in relation to the sham group and a significant increase in the values of the parameters BS/BV and Tb.Sp compared to the OVX group. In the case of the SrC group, the administration of SrC contributed to a decrease in BV/TV and Conn.Dn in relation to the sham group and a significant increase in BS/BV in relation to the OVX group. Supplementation with SrCl contributed to a significant decrease in the value of Conn.Dn compared to the sham group. However, comparing the SrCl and OVX groups, the statistical analysis showed a significant increase in BS/BV (Figure 4). BV/TV was significantly reduced in the OVX + SrC group compared to those receiving SrCl (Figure 4). The representative micro-CT images of the 3D reconstruction and the transverse plane of the cortical femurs of mice with induced osteoporosis after SrR, SrC, and SrCl administration are shown in Figure 5.

### 2.5. Cortical Bone Morphology

The statistical study between the OVX and sham groups showed an increase in MMI(y) in sham-treated mice compared to OVX-treated mice. The value of Gr.R(z) and Gr.R(polar) was significantly lower in the OVX group compared to the sham group. 

**Figure 6:**
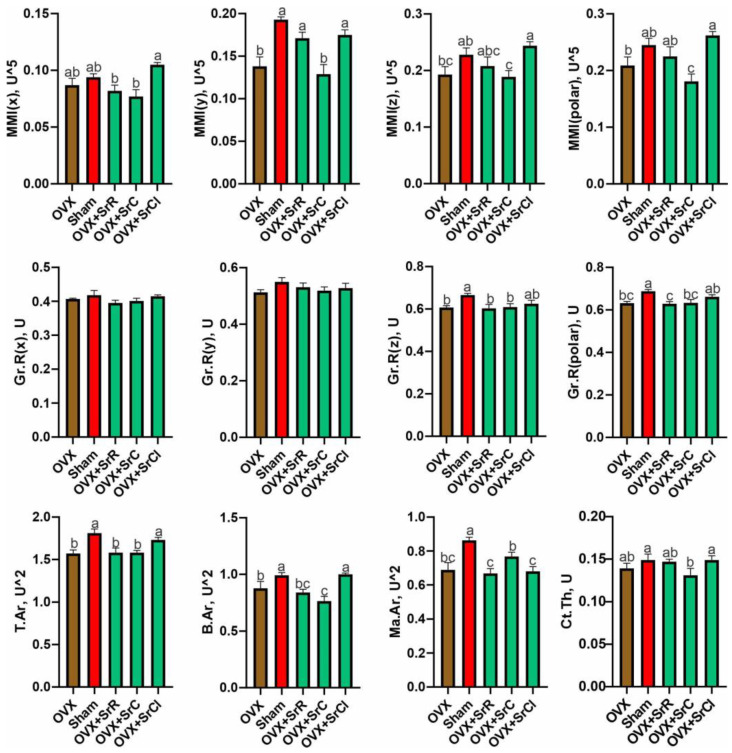
Effect of SrR, SrC, and SrCl administration on the cortical bone morphology of the femur in mice with induced osteoporosis, calculated based on micro-CT images. The data given are the mean (n = 10) with the standard error. The values with different letters differ significantly at *p* < 0.05. OVX—ovariectomy group without supplementation; sham—sham ovariectomy group without supplementation; OVX + SrR—ovariectomy + strontium ranelate; OVX + SrC—ovariectomy + strontium citrate; OVX + SrCl—ovariectomy + strontium chloride. MMI(x)—moment of inertia (x); MMI(y)—moment of inertia (y); MMI(z)—moment of inertia (z); MMI(polar)—polar moment of inertia; Gr.R(x)—radius of gyration (x); Gr.R(y)—radius of gyration (y); Gr.R(z)—radius of gyration (z); Gr.R(polar)—polar radius of gyration.; T.Ar—mean total cross-sectional tissue area; B.Ar—mean total cross-sectional bone area; Ma.Ar—medullary (or marrow) area, Ct.Th—average cortical thickness.

Administration of SrC contributes to a significant decrease in the MMI(y), MMI(z), and MMI(polar) values compared to the sham group. On the other hand, mice from the OVX + SrR and OVX + SrCl groups had higher MMI(y) values than the OVX group. Individuals from the SrCl group had significantly higher MMI(z) and MMI(polar) values compared to animals from the OVX group. In the case of Gr.R(z) and Gr.R(polar), a decrease in the values of these parameters was observed in the OVX + SrR and OVX + SrC groups compared to the sham group (Figure 6).

The OVX group supplemented with SrCl was characterized by the highest MMI(x) value among the other groups receiving strontium salts. On the other hand, MMI(y) and MMI(polar) were significantly lower in animals from the OVX + SrC group compared to other experimental groups. In the cases of MMI(z) and MMI(polar), significant differences were observed between the OVX + SrC and OVX + SrCl groups. The OVX group receiving SrCl had a significantly higher value of this parameter. Gr.R(polar) was significantly higher in the OVX + SrCl group compared to the SrR group (Figure 6).

The 2D analysis of the cortical bone of the femurs of experimental animals performed using micro-CT showed a significant decrease in the values of T.Ar, B.Ar, and Ma. Ar in animals from the OVX group compared to the sham group. However, comparing both the OVX and sham groups to the experimental groups, a reduction of T.Ar, B.Ar, and Ma. As was observed in the OVX + SrR and OVX + SrC groups compared to the sham group. Also, Ma. Ar was reduced in the OVX + SrCl group. In addition, a significant decrease in Ct. was observed in the OVX + SrC group receiving SrC compared to the sham group. However, compared to the OVX group, the OVX + SrCl group was characterized by a higher value of T.Ar and B.Ar. On the other hand, in the OVX + SrC group, a reduction in B.Ar was observed compared to animals from the OVX group. However, comparing the experimental groups with each other, the 2D analysis of the femoral cortex showed a significantly higher value of T.Ar and B.Ar in the OVX + SrCl group compared to the other groups receiving strontium salts. In the case of Ma. Ar, the OVX + SrC group was characterized by the highest value of this parameter from all experimental groups. Also, in the OVX + SrC group, supplementation with the strontium salt contributed to a significant reduction of Ct. compared to the OVX + SrCl group (Figure 6).

### 2.6. Content of Ca, P, and Sr in the Mineral Fraction of Bone Tissue

**Figure 7:**
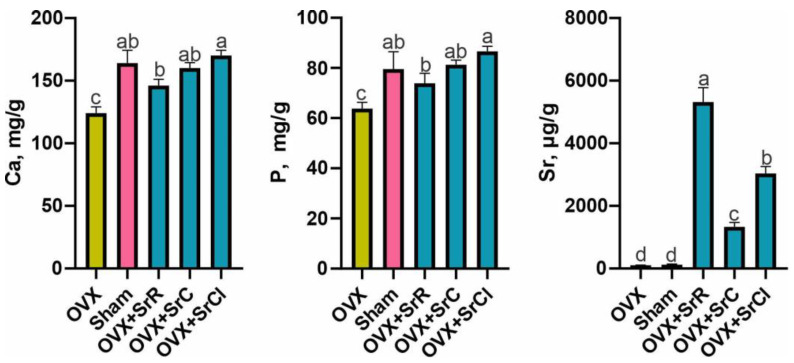
Effect of SrR, SrC, and SrCl administration on the content of Ca, P, and Sr in the bone tissue of the femur in mice with induced osteoporosis. The data given are the mean (n = 10) with the standard error. The values with different letters differ significantly at *p*<0.05. OVX—ovariectomy group without supplementation; sham—sham ovariectomy group without supplementation; OVX + SrR—ovariectomy + strontium ranelate; OVX + SrC—ovariectomy + strontium citrate; OVX + SrCl—ovariectomy + strontium chloride.

The analysis of the composition of the mineral fraction of the bone tissue of the femur showed that the animals from the OVX group had a significantly lower calcium and phosphorus content in comparison to the animals from the sham group. Sr content was not significantly different in either group (Figure 7).

All groups receiving Sr were characterized by a significantly higher content of Ca and P compared to the OVX group. Such differences were not observed between the sham group and groups OVX + SrR, OVX + SrC, and OVX + SrCl. In the case of Sr content, it was shown that all groups receiving strontium salts had a significantly higher content of this element in the mineral fraction compared to both controls (Figure 7).

The OVX group receiving SrCl was characterized by a higher share of Ca and P in relation to the OVX + SrR group. However, in the case of Sr, the OVX group receiving SrR had the highest Sr content in the mineral fraction compared to the other experimental groups. The OVX + SrC group had the lowest content of this element in relation to the OVX groups receiving SrR and SrCl (Figure 7).

## 3. Discussion

Strontium in the treatment of osteoporosis is administered in the form of a salt. One of the most studied salts is strontium ranelate (SrR), a drug used, in particular, in the treatment of postmenopausal osteoporosis. Its effectiveness lies in its dual action, including increasing bone formation and inhibiting bone resorption [15]. These effects have been confirmed by many studies, both on animal models as well as in clinical trials conducted with the participation of humans [16,17,18]. Since 2014, the use of SrR has been restricted due to reports of side effects, mainly an increased risk of cardiovascular complications [14]. Therefore, many limitations have been applied to the treatment of osteoporosis with SrR, which contributed to the search for other options in the administration of strontium, e.g., as strontium chloride (SrCl) or strontium citrate (SrC) [19,20,21]. However, there are only a few studies evaluating the effect of these salts on bone tissue [11,22,23,24,25,26]. Furthermore, there is a lack of work comparing these three salts in an animal model with induced osteoporosis. Therefore, in the presented work, the effect of these strontium salts on bone tissue was investigated using an ovariectomy (OVX)-induced osteoporosis mouse model. In the presented study, supplementation lasted 16 weeks.

The study showed that after 16 weeks of administration of strontium salts, the body weight of the tested animals was not significantly different from the OVX group. The lack of effect of SrR on animal body weights was also observed in numerous studies that were carried out in various experimental models and various animal species, including mice, rats, young hens, and monkeys in growing animals and in individuals, in which osteoporosis was induced [10,16,18,23,27,28,29,30]. Also, no effect on body weight was noted in the experiment with strontium carbonate (SrCO_3_) using young hens [31] and rats [32]. In turn, in a two year study by Delannoy’s team, no changes in body weight were observed, regardless of the SrR dose, in the first year. However, in the second year, a slight increase in body weight in mice receiving 7.01 mmol Sr/kg/d was observed compared to the control group. Nevertheless, at the end of the experiment, only differences between the sexes were observed without the influence of different doses of SrR [16]. Lack of changes in body weight were also noted in studies involving SrC and SrCl. Grynpas and Marie (1990) did not record an increase in animal weight after supplementation of SrCl [22]. Also, Marie and Hott conducted a short-term supplementation experiment with SrCl that did not significantly change the body weight of the animals [23]. A study by Taylor et al. from 2017 on a rabbit model of MDO, in which animals received SrC, showed that all rabbits gained weight correctly during the study [24].

To the best of our knowledge, there are no studies evaluating the effect of SrC and SrCl supplementation on animal body weight in mice with induced osteoporosis. Based on the studies briefly described above, it could be perceived that Sr, regardless of the dose, age, sex, duration of supplementation, and experimental system, does not affect body weight. However, the addition of other elements like Ca or the inclusion of exercises was associated with a decrease in the body weight in the experimental groups [33,34].

In our study, at the end of the experiment, animals in the sham group were lighter than in all other experimental groups (SrR, SrC, and SrCl) and the OVX group. Such an effect was also observed in the literature [18,23,34], confirming that OVX and sham surgeries were correctly performed.

The analysis of osteometric properties, including bone mass and length, showed no impact of SrR, SrC, and SrCl supplementation on the femur weight of the tested animals. Additionally, SrC and SrR also did not affect the femur length and the other researchers also did not observe any effect of SrR on this parameter [17,34]. However, we observed a slight but statistically significant increase in femur length among animals in the SrCl group compared to the OVX and sham groups. In contrast to our findings, Marie et al. reported that an 8 week supplementation with SrCl did not significantly affect tibia length in 21-day-old growing mice [35], which may be attributed to the shorter duration of SrCl treatment in their experiments. On the other hand, the administration of strontium salts led to a significant increase in trabecular and cortical tissue mineral density (TMD) in all experimental groups. Among the Sr groups, the OVX + SrC group exhibited the smallest increase in TMD. These results may indicate that the administration of strontium significantly increases the mineral density of bone tissue, which is independent of bone mass, as it is converted into surface area. Although some other studies have shown that Sr is more strongly incorporated into the trabecular bone compared to cortical bone [20,36,37,38], in our study, TMD increased similarly in both the cortical and trabecular bones. Specifically, in the trabeculae, TMD increased by 36.9%, 16.5%, and 44.0% in the OVX + SrR, OVX + SrC, and OVX + SrCl groups, respectively, compared to the OVX control. In the case of compact bones, TMD increased by 35.6%, 22.6%, and 40.0%, respectively. These results are consistent with the findings of other researchers [39,40,41,42,43]. Our results revealed a positive correlation between the content of strontium in bone and TMD in both trabecular and cortical tissue. Additionally, a positive correlation was observed in old pullets supplemented with strontium for 11 months for bone mineral density and bone mineral content [31].

In animal studies, OVX has been shown to reduce both BMC and BMD in the whole body. However, an 8 week administration of SrR eliminates this effect [33]. Similarly, long-term studies have demonstrated that SrR treatment increases BMD in rats [27,29]. Short-term administration of SrR (for 10 days) has also been shown to reduce BMD loss induced by immobilization and to improve bone mineral density in a previously immobilized limb [17].

All the mentioned studies discussed the BMD of the entire examined bone without distinguishing between cortical and cancellous bone tissue. Furthermore, they exclusively focused on the impact of SrR administration. In contrast, our research delves into greater detail by separately assessing the effects of SrR on trabecular and cortical bone.

The available literature lacks studies evaluating the effect of SrC and SrCl on bone tissue BMD. Our results indicate that both of these supplements improve the TMD of both types of bone tissue. However, SrC appears to have the least effective impact on bones, in the case of both trabecular and cortical bones.

Only a few studies have described the effect of SrC on BMD. An increase in BMD was observed in women diagnosed with osteoporosis who took SrC for two years at a dose of 680 mg; however, the research group was very small (n = 3) [25]. Furthermore, a significant increase in BMD in the entire zebrafish skeleton was reported following 12 weeks of supplementation with SrC [26]. On the other hand, in contrast to our findings, no effect of SrCl administration for 8 weeks on the BMD of healthy animals was observed [22].

The analysis of the microarchitecture of the bone trabeculae conducted in our work showed that the administration of SrR, SrC, and SrCl significantly affects the growth of BS and BS/BV. We observed a significantly higher BS value in the group receiving Sr compared to the control. Administration of strontium chloride to animals after OVX contributed to an increase in BS/TV. In the case of the SrC group, strontium affected the increase in TV and SrR treatment, increasing Tb.Sp. Comparing the group after OVX to the group that underwent the sham OVX procedure, it can be observed that there was a reduction in some histomorphometry parameters of cancellous bone tissue as a result of the bilateral removal of the ovaries. However, this effect was not substantial but may be related to the fact that ovariectomy was performed in young animals, in which osteogenesis is still dominant over osteolysis. Based on our results, the young age of the animals may have contributed to the reduction in the ovariectomy effect, as there could still be a higher compensation for the loss of bone mass as a result of increased osteogenesis processes at this age [32,44,45].

Analysis of hip bone biopsies obtained from the STRATOS (STRontium Administration for Treatment of OSteoporosis), SOTI (Spinal Osteoporosis Therapeutical Intervention), or TROPOS (TReatment of Peripheral OSteoporosis) trials showed an improvement in histomorphometric parameters as a result of SrR treatment. An increase in the number of bone trabeculae and a reduction in the distance between the trabeculae, as well as a reduction in the structural model index were noted [46]. Also, the increase in the number of trabeculae and the decrease in the spacing between the trabeculae were observed in a study by Rizzoli et al. [47]. However, in the study by Chavassieux et al., which was a multicenter, double-blind, international trial that used biopsies of hip bones from OP women treated with SrR 2.0 g/day (n = 256) or alendronate 70 mg/week (n = 131), different results were obtained. Treatment with SrR for 12 months contributed to the significant reduction in some trabecular bone parameters (the number of nodes/tissue volume, trabecular bone volume, trabecular thickness, and number) [48]. This study showed no significant influence on the process of osteogenesis. In our study, no changes in these parameters were observed, regardless of the administered strontium salts.

Currently, the available literature discusses whether strontium, especially in the form of SrR, has an anabolic effect, which can be observed in the improvement of histomorphometric parameters. In animal studies, the microarchitecture of the bone trabeculae is often improved. However, the doses used are higher than those taken by humans during therapy with this drug [49].

The morphological parameters obtained in our study show that the administration of strontium salts contributes minimally to compensating for the effects of sham ovariectomy. Also, there were no large differences between the OVX and sham groups. The lack of noticeable changes may be due to the young age of the animals.

Strontium, like calcium, is incorporated into bones [20,38]. Numerous studies report that the amount of strontium deposited in bones depends on factors such as dosage, sex, and calcium intake [10,50]. In our study, we assessed the strontium content in the mineral portion of bone tissue. Administration of each of the strontium salts led to an increase in the Sr content of the bone’s mineral portion. Notably, our findings indicate that SrR resulted in the highest Sr concentration among all the salts, while the lowest concentration was observed following SrC administration, consistent with the findings of Pemmer et al. [51]. In contrast, Wohl et al., who examined both SrC and SrR, reported that both salts delivered an equivalent amount of Sr to the bone. Our results differ from these findings, which could be attributed to differences in experimental setups. In the study by Wohl et al., strontium was administered to growing 18-week-old rats for 10 weeks [7], whereas in our experiment, 7-week-old mice with induced osteoporosis were used and salt administration continued for 16 weeks. Additionally, different Sr doses were employed in the experiments.

Furthermore, we determined the calcium (Ca) and phosphorus (P) content in the mineral portion of the bone tissue. Our experiment revealed that all groups receiving Sr exhibited significantly higher Ca and P levels compared to the OVX group. When comparing the experimental groups, the SrCl group displayed a higher concentration of Ca and P in relation to the other group.

The strength of our work is the wide use of micro-CT examination, which allows us to obtain many results during one analysis and to thoroughly examine the bones. Furthermore, we were the first to conduct a study in an animal model of induced osteoporosis to assess the effect of SrR, SrC, and SrCl on changing osteometric properties, BMD, bone morphology, and microarchitecture. A strong element of our work is the selection of an appropriate time for the administration of strontium salts. It has been proven in the literature that 16 weeks is the time when the greatest changes in bone occur as a result of OVX. Research conducted by Wronski et al. [52] showed that more than 150 days after the OVX procedure, osteoblast surfaces and osteoclast surfaces in ovariectomized rats decreased to the levels of cells taken from the sham ovariectomy group. As well, micro-CT studies have shown that the most intense changes in bone tissue after OVX treatment occur between 8 and 16 weeks after surgery [15,18,52].

The strength of our work also lies in the separate determination of TMD for the trabeculae and cortical bone studies. There are also no studies evaluating whether the administration of molar equivalent doses of SrR, SrC, and SrCl delivers the same amount of Sr to the bone and whether the amount of Sr affects the content of Ca and P in the bone. It should also be noted that this is the first study to examine the effect of SrC and SrCl supplementation on the parameters of the microarchitecture of bone trabeculae in animals with induced osteoporosis. Therefore, further research in this area is required and our results differ from those reported by other authors.

**Study Limitations:** The experimental conditions differ from the clinical conditions in which osteoporosis is most often diagnosed. Supplementation was started immediately after the OVX procedure without leading to bone loss. In clinical conditions, patients are referred for treatment at the time of significant BMD loss and often report to osteoporosis clinics after low-energy fractures. In our study, serum estrogen concentrations were not determined to confirm a properly performed OVX procedure. The assessment of the correctness of the procedure was carried out post-mortem. Furthermore, this study does not allow for the assessment of the impact of supplemented strontium salts on the processes of osteolysis and osteogenesis, due to the lack of evaluation of bone turnover markers. Moreover, no mechanistic analysis has been carried out.

The mentioned issues are the foundations for further research and a broader assessment of strontium salts including their impact on markers of bone turnover and the strength properties of bone tissue.

## 4. Materials and Methods

### 4.1. Animals and Experimental Design

The experiment was approved by the Local Ethical Committee No. 1 in Lublin at the Medical University (Resolution No. Nr 35/2015). The place of the experiment was the Experimental Medicine Center of the Medical University of Lublin. All individual stages of the experiment were carried out following the requirements included in the resolution. The various stages of the study were carried out in such a way as to minimize the stress and suffering of the animals. The experiment was conducted in accordance with ARRIVE guidelines and all methods were performed in accordance with the relevant guidelines and regulations.

Fifty-five-week-old female SWISS mice weighing 18–19 g were used in the experiment. All animals were kept in separate cages and acclimated for 14 days. Throughout the experiment the animals were kept in standard laboratory conditions, as follows: 12/12 h light cycle, a room temperature of 21 ± 3 °C, and a humidity of 55 ± 5%. All animals were fed ad libitum standard laboratory animal feed and had free access to fresh water [53].

After quarantine, healthy animals were weighed and were randomly classified into one of the following five groups: OVX, sham, OVX + SrR, OVX + CV, and OVX + Cl. Each group consisted of 10 animals. In 40 females from groups OVX + SrR, OVX + C, and OVX + Cl, ovariectomy (OXV) was performed via the bilateral removal of the ovaries to induce osteoporosis [54]. In total, 10 females from the sham group underwent a sham ovariectomy (sham). During sham surgery, the animals underwent a similar procedure, except for the removal of the ovaries [55,56].

All procedures were performed under anesthesia. Premedication was given prior to anesthesia. Dexmedetomidine, butorphanol, and sevoflurane were used for anesthesia and premedication, according to the following schedule: dexmedetomidine at a dose of 0.5 mg/kg body weight was used for premedication. s.c., butorphanol at a dose of 3 mg/kg b.w. s.c., and inhaled sevoflurane, 2–3% during induction, and 1.5–2% for maintenance of the anesthesia. Meloxicam at a dose of 2 mg/kg body weight was used to relieve pain. s.c. All used drugs were previously recommended by veterinarians. The animals were monitored during the perioperative and postoperative periods.

After surgery, the animals were placed separately. The first two groups, OVX and sham, received clean drinking water. Groups III (SrR Group), IV (SrC Group), and V (SrCl Group) received strontium ranelate, strontium citrate, and strontium chloride in drinking water at doses corresponding to 7.5 mmol/L strontium concentration [53].

During the experiment, the weight of the animals was checked every 2 weeks from the day of the OVX or sham procedure. The weight of the animals was monitored using an electronic scale. On the other hand, the amount of water and feed consumed was checked daily. There were no significant differences between the studied groups in the amount of water and food consumed.

After 16 weeks of the experiment, the animals were euthanized by decapitation [55]. Before decapitation, the animals were anesthetized. Postmortem mice from the OVX group were found to have successfully undergone ovariectomy due to a lack of ovarian tissue and atrophy of the uterine horn. The research material consisted of right femur tissue. The bones were cleaned of soft tissues, weighed, measured, and frozen at a temperature of −80 °C for further analysis.

### 4.2. Administration of SrR, SrC, and SrCl

SrR, SrC, and SrCl were administered immediately after ovariectomy in the drinking solution. Fresh drinking solutions were provided every two days. SrR, SrC, and SrCl were dissolved in 500 mL of distilled water to obtain solutions with equivalent molar concentrations of strontium 7.5 mmol/l [48].

### 4.3. Miro-CT Analysis

The study of the structural properties was carried out with the use of a SkySkan 1172 computer microtomograph, Bruker (Koenitch, Belgium). In order to determine the values of the structural parameters, the research was carried out in the following stages: registration of femoral samples; reconstruction of samples using NRecon, Bruker software ver. 2.0; preparation of representative bone volumes (VOI) for measuring the structural properties of bone and dense tissue; and measurement of 3D structural parameters using CTAn, Bruker software.

**Figure 8:**
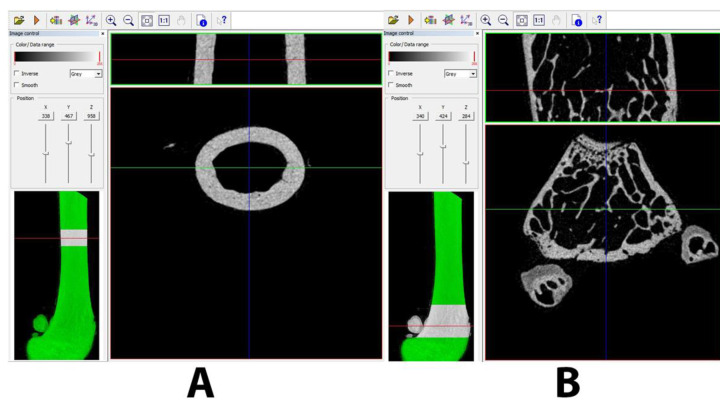
Representative photo of the selected sample volume (VOI), representing cancellous tissue (**A**) and cortical tissue (**B**). The VOI contained the following number of cross-sections for cortical bone tissue: 252, and for cancellous tissue: 127.

Each of the samples was recorded with a resolution of 6 µm, using the following lamp parameter: 59 kV/169 uA. Additionally, a 0.5 mm Al filter was used. The single exposure time was 800 ms, the total recording angle was 180 degrees, and the unit rotation angle was 0.52 degrees. To measure the structural parameters characterizing the cancellous and cortical bone tissues, two volumes (VOI) were separated from each bone. The number of sections for the volume of VOI for cancellous bone tissue is 252, which corresponds to a height of 1.5 mm, while for cortical tissue, the number is 127 sections. The selected areas for the analysis of structural parameters are shown in Figure 8. The selection method and selection of the areas of analysis were chosen under the previously proposed guidelines [13,14].

Using the analysis described above, the following morphometric and morphological parameters of the cancellous and cortical bones of the femur were determined: For cortical bones, TMD and morphological properties, such as MMI(x)—moment of inertia (x); MMI(y)—moment of inertia (y); MMI(z)—moment of inertia (z); MMI (polar)—polar moment of inertia; Gr.R(x)—radius of gyration (x); Gr.R(y)—radius of gyration (y); Gr.R(z)—radius of gyration (z); Gr.R(polar)—polar radius of gyration; T.Ar—mean total cross-sectional tissue area; B.Ar—mean total cross-sectional bone area; Ma.Ar—medullary (or marrow) area, Ct.Th—average cortical thickness, were determined.

In the case of trabecular bones, BS/BV—specific bone surface; BS/TV—bone surface density; BV/TV—percent bone volume; BS/TV—bone surface density; Tb.Th—trabecular thickness; SMI—structure model index; FD—fractal dimension; Conn.Dn—connectivity density, were determined.

### 4.4. Analysis of the Content of Calcium, Phosphorus, and Strontium in the Mineral Fraction of Bone Tissue

After the micro-CT analysis, whole femurs were used to determine the content of calcium, phosphorus, and strontium in the mineral fraction of the bone tissue. The bones were powdered and the dry samples were digested in a mixture of HNO_3_ and H_2_O (2:8 *v*/*v*). The procedure was performed in a microwave digester (TOPwave, Analytic Jena AG, Jena, Germany). The conical dilution had a volume of 25 mL. The quantification of Ca, P, and Sr was carried out using ICP-OES PlasmaQuant PQ 9000 Elite (Jena AG Analyst, Jena, Germany). The entire analysis has been described previously [57].

### 4.5. Statistical Analysis

All results are presented as mean values with standard error of the means (mean ± SEM). Before performing the significance tests, it was checked whether the examined features were normally distributed using the W. Shapiro–Wilk test and the homogeneity of variance using the Levene test. After this, the results were statistically analyzed using a one-way analysis of variance, while the difference between the objects was determined with Tukey’s test at the 0.05 probability level. All calculations were conducted using Statistca ver. 13.3 (TIBCO Software Inc. 2017).

## Data Availability

The data presented in this study are available on request from the corresponding author.
